# Synergistic inhibition of pneumococcal growth by *Dolosigranulum pigrum* and *Corynebacterium pseudodiphtheriticum:* insights into nasopharyngeal microbial interactions

**DOI:** 10.1128/spectrum.00138-25

**Published:** 2025-05-30

**Authors:** M. Cisneros, M. Blanco-Fuertes, A. Lluansí, P. Brotons, D. Henares, A. Pérez-Argüello, G. González- Comino, P. Ciruela, A. Mira, C. Muñoz-Almagro

**Affiliations:** 1 Grup de Recerca, Institut de Recerca Sant Joan de Déu (IRSJD), Hospital Sant Joan de Déu 16512 , Barcelona, Catalonia, Spain; 2 Faculty of Medicine and Health Sciences, Universitat Internacional de Catalunya 120014 , Sant Cugat del Vallès, Catalonia, Spain; 3 CIBER de Epidemiología y Salud Pública (CIBERESP), Instituto de Salud Carlos III 38176, Madrid, Community of Madrid, Spain; 4 Agència de Salut Pública de Catalunya 535551, Barcelona, Catalonia, Spain; 5 Genomics and Health Department, FISABIO Foundation 203353 , Valencia, Valencian Community, Spain; Children's National Hospital, George Washington University, Washington, DC, USA

**Keywords:** *Streptococcus pneumoniae*, *Dolosigranulum pigrum*, *Corynebacterium pseudodiphtheriticum*, invasive pneumococcal disease, capsular serotype, nasopharyngeal microbiota

## Abstract

*Streptococcus pneumoniae* is a nasopharynx colonizer that can invade sterile tissues, causing invasive pneumococcal disease (IPD). *Dolosigranulum pigrum* and *Corynebacterium pseudodiphtheriticum* are commensal bacteria commonly isolated from the nasopharynx of healthy children, potentially playing a protective role. This study aims to analyze the effects of *D. pigrum* and *C. pseudodiphtheriticum* on *S. pneumoniae in vitro* growth. Pneumococcal strains were collected from IPD patients and healthy carriers in Catalonia (2016–2023). *D. pigrum* and *C. pseudodiphtheriticum* strains were isolated from a healthy child’s nasopharynx. *S. pneumoniae* was co-cultured with each commensal bacterium in triplicate experiments. Pneumococcal growth was quantified using a real-time PCR assay targeting the *lytA* gene. The effect of commensal bacteria on pneumococcal growth was evaluated using a linear mixed-effect regression model. Twenty-eight pneumococcal strains expressing 24 different serotypes and 27 clonal types were analyzed (18 isolated in blood and 10 in nasopharyngeal aspirate). Pneumococcal growth was decreased by *D. pigrum* (β = −0.763, 95% confidence interval [CI]: −0.94 to −0.59, *P* < 0.0001) and *C. pseudodiphtheriticum* (β = −0.583, 95% CI: −0.76 to −0.41, *P* < 0.0001). The combined presence of both had a stronger inhibitory effect (β = −0.971, 95% CI: −1.15 to −0.79, *P* < 0.0001). No association was found between isolation site or serotype with pneumococcal growth. *D. pigrum* and *C. pseudodiphtheriticum* significantly reduced pneumococcal growth, with a synergistic effect when combined. This antagonistic effect supports the potential protective factor of healthy nasopharyngeal microbiota against IPD and the development of these microorganisms as probiotics.

**IMPORTANCE:**

Invasive pneumococcal disease (IPD) is a significant worldwide health challenge. The present study highlights the significant inhibitory effect of two commensal bacteria, *Dolosigranulum pigrum* and *Corynebacterium pseudodiphtheriticum*, on pneumococcal growth, with a stronger effect observed when both bacteria are present together. Through testing different strains of *S. pneumoniae* and the implementation of a robust statistical model, this research advances in the knowledge of microbial ecology and provides evidence to support the development of the use of these commensal bacteria as probiotics. These results emphasize the possibility of using the nasopharyngeal microbiota’s natural interactions to mitigate the risk of IPD.

## INTRODUCTION

*Streptococcus pneumoniae* is a Gram-positive bacterium that asymptomatically colonizes the human nasopharynx, especially among children aged under 5 years. However, under certain circumstances, it has the potential to invade sterile tissues and cause disease (1). Invasive pneumococcal disease (IPD) is the most severe form of pneumococcal infection and includes bacteremia, pneumonia, meningitis, and septicemia, among others. Despite the availability of pneumococcal vaccines, it has been estimated that *S. pneumoniae* is responsible for over 300,000 deaths annually in children under 5 years of age (2).

Pneumococcal conjugate vaccines (PCVs) are developed against the bacterium’s main virulence factor, the capsular polysaccharide (CPS) (3). There are more than 100 known capsular serotypes with different degrees of invasiveness; some of them are high invasive disease potential serotypes (4–6). Following the introduction of conjugate vaccines, numerous studies have shown a decreased prevalence of vaccine serotypes (VTs) and a significant reduction in IPD caused by these serotypes. Nevertheless, vaccines do not cover all serotypes, leading to the phenomenon of serotype replacement, in which non-vaccine serotypes (NVTs) are expanded and become more prevalent in disease (7, 8).

Pneumococcal nasopharyngeal colonization represents a key factor to understand the burden of pneumococcal disease and address its prevention. Colonization is a complex and dynamic process in which multiple factors interplay, including environmental, host, and microbiota factors. Intricate interactions with other common inhabitants of the nasopharynx seem to be an important step for pneumococcal colonization (1, 9).

Previous studies have suggested that certain natural colonisers of the nasopharynx could play a protective role against the colonization of *S. pneumoniae* (10). Significant abundances of *Dolosigranulum pigrum* and *Corynebacterium* species have been found in nasal and nasopharyngeal microbiota of children when *S. pneumoniae* is absent (10–12). Furthermore, increased abundances have been detected in healthy controls compared with patients with different respiratory infections, suggesting a potential beneficial role in respiratory health through negative interactions with common pathobionts (10–19). In line with these microbiota studies, it has been observed that *D. pigrum* appears to modulate respiratory innate immunity and enhance resistance to pathogens, such as respiratory Syncytial virus (RSV) and *S. pneumoniae (16*). Notably, nasal administration of *D. pigrum* in mice has been shown to decrease the number of *S. pneumoniae* and reduce its spread to the blood (15, 18).

*D. pigrum* is one of the known species of the genus *Dolosigranulum* that was first reported (20, 21). In terms of structure, it is a Gram-positive coccus arranged in pairs, tetrads, and clusters. This catalase-negative bacterium is generally sensitive to antibiotics, such as beta-lactams (22). A notable characteristic of *D. pigrum* that enhances it as a potential protective species is its ability to produce lactate, classifying it as lactic acid bacterium (LAB) (23, 24). Additionally, genome sequencing has confirmed this classification by identifying genes associated with the homofermentation of carbohydrates (24, 25). Most LABs have been found in the digestive tract, where they perform beneficial tasks for human health, such as enhancing resistance to pathogens through microbe-microbe interactions, the synthesis of bacteriocins, or immunomodulation (26). For instance, *Lactobacillus murinus,* a LAB from the lung microbiota in mice, can inhibit *S. pneumoniae* growth *in vitro* and prevent colonization *in vivo* (27).

*D. pigrum* is commonly isolated from the nasal cavity or the nasopharynx, where it coexists with other commensal bacteria such as *Corynebacterium spp.* (11). It has also been described that *Corynebacterium pseudodiphtheriticum*, one of the species of this genus, could also prevent pathogenic bacteria colonisation in the nasopharynx in cooperation with *D. pigrum* (28). Brugger et al. demonstrated increased relative abundances of *C. pseudodiphtheriticum* were increased in the presence of *D. pigrum*. Additionally, they observed that *C. pseudodiphtheriticum* could enhance the growth of *D. pigrum in vitro,* supporting its beneficial effect (14).

Despite this extensive evidence of the potential protective role and these suggestive results, little is known about the impact of *D. pigrum* and *C. pseudodiphtheriticum* on pneumococcal replication rate and the consistency of these results according to different capsular serotypes of *S. pneumoniae*. Thus, the aim of the present study is to analyze the effects of the two commensal bacteria, *D. pigrum* and *C. pseudodiphtheriticum,* on *Streptococcus pneumoniae in vitro* growth (IVG) in a diverse collection of *S. pneumoniae* strains.

## MATERIAL AND METHODS

### Bacterial strain collections

Invasive pneumococcal strains were obtained from blood samples of all ages from the collection of the Support Laboratory for Molecular Epidemiology of IPD in Catalonia during 2016–2023. Isolates were identified at the hospital of origin by standard microbiological techniques such as Gram staining, culturing requirements, colony morphology, optochin sensitivity testing, and bile solubility test. After presumptive confirmation, the strains were sent to the Molecular Microbiology Department of the University Hospital of Sant Joan de Déu for epidemiological surveillance of *S. pneumoniae*. Upon reception of the isolates, multiplex PCR and sequencing analysis were performed to determine the identification of the capsular pneumococcal serotypes and clonal type (29). Carriage strains (*D. pigrum, C. pseudodiphtheriticum,* and *S. pneumoniae*) were prospectively collected from nasopharyngeal aspirate (NPA) samples with informed consent in the framework of two consecutive funded projects focused on the role of nasopharyngeal microbiota in respiratory health and disease during 2016–2023. Especially, the commensal bacteria strains were isolated from an NPA sample of healthy children who attended Sant Joan de Déu Barcelona Children’s Hospital (SJD) in autumn of 2018. All the strains were preserved in 1 mL of preservation media of skim milk at −80°C. The 28 pneumococcal strains were selected to represent the main serotypes and clones detected in Catalonia during the study period as reported on the Microbiological Notification System of Catalonia. Selection criteria were based on epidemiological importance, particularly prevalence, invasiveness level, and representation of the most prevalent serotypes circulating in the region in different age groups.

### Isolation and identification process of *Dolosigranulum pigrum* and *Corynebacterium pseudodiphtheriticum*

Isolation of both bacteria was performed by culturing 100 µL of NPA sample on blood agar plates (Columbia agar supplemented with 5% sheep blood; BioMérieux). Additionally, *D. pigrum* was isolated on CNA Agar plates (Columbia CNA agar supplemented with 5% sheep blood; Becton Dickinson) and mannitol salt agar (Becton Dickinson). The plates were incubated at 37°C under aerobic conditions supplemented with 5% CO_2_.

Matrix-assisted laser desorption/ionization time-of-flight (MALDI-TOF) mass spectrometry was used to identify both commensal bacteria. This system generates a spectrum based on the mass-charge relationship of the microorganism’s proteins, which is then compared with reference libraries containing the different spectra of known microorganisms (30).

### Isolation, identification, and molecular characterization process of *Streptococcus pneumoniae*

To isolate *S. pneumoniae,* 100 µL of the NPAs were directly cultured in Blood Agar plates (Columbia agar supplemented with 5% sheep blood; BioMérieux) and incubated at 37°C under aerobic conditions supplemented with 5% CO^2^. Pneumococcal identification was conducted using standard microbiological techniques, such as the optochin sensitivity test and colony morphology (31). In addition, capsular typing was performed on all pneumococcal isolates using fluorescence fragment analysis and Whole Genome Sequencing (WGS) (32, 33). According to previous literature (5), serotypes 1, 3, 4, 5, 7F, 8, 9A, 9V, 12F, 14, 18, 19A, 24F, and 33F were considered high invasive disease potential serotypes (HIPST), while the rest were considered low invasive disease potential serotypes (LIPST).

### *In vitro* growth of pneumococcal isolates according to *D. pigrum* and *C. pseudodiphtheriticum* exposure

#### Preparation and standardization of *D. pigrum-* and *C. pseudodiphtheriticum-*enriched media

**Fig 1:**
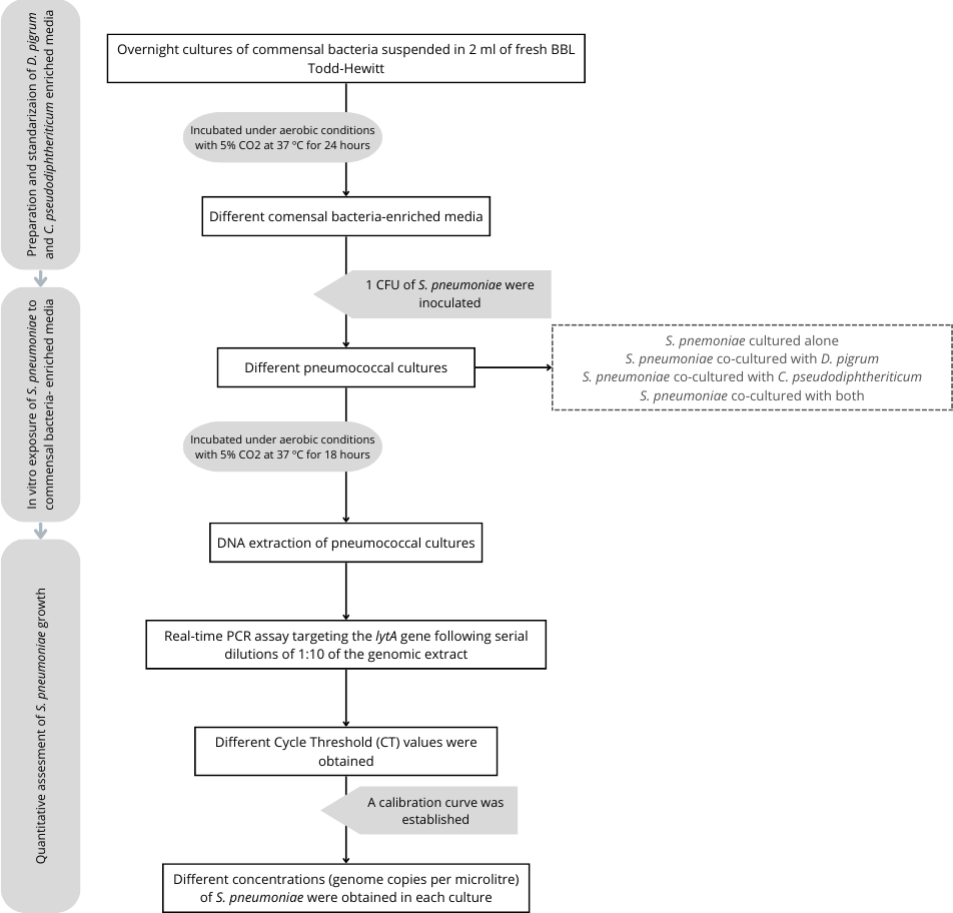
Flow diagram of the protocol for *in vitro* growth of pneumococcal isolates according *to D. pigrum* and *C. pseudodiphtheriticum* exposure.

Isolates of *D. pigrum* and *C. pseudodiphtheriticum* preserved at −80°C were cultured on blood agar plates (Columbia agar supplemented with 5% sheep blood; BioMérieux). To establish different *in vitro* conditions for *S. pneumoniae*, overnight cultures of *D. pigrum* and *C. pseudodiphtheriticum* (grown in aerobic conditions with 5% CO^2^ at 37°C) were suspended in 2 mL of fresh BBL Todd-Hewitt broth (Becton Dickinson). The suspension solution was adjusted to a concentration of 6 log_10_ genome copies (gc)/µL ± 0.7 log_10_, determined by Qubit Fluorometric Quantification from the extraction of genomic DNA (Fig. 1)*.*

#### *In vitro* exposure of *S. pneumoniae* to commensal bacteria-enriched media

In order to expose *S. pneumoniae* to commensal bacteria, 1 CFU of each pneumococcal isolate was inoculated into four culture conditions, resulting in the following pneumococcal broth cultures: (i) *S. pneumoniae* cultured alone in 2 mL of BBL Todd-Hewitt broth (Becton Dickinson) as negative controls (S); (ii) *S. pneumoniae* co-cultured with *D. pigrum* (SD); (iii) *S. pneumoniae* co-cultured with *C. pseudodiphtheriticum* (SC); and (iv) *S. pneumoniae* co-cultured with both commensal bacteria, *D. pigrum* and *C. pseudodiphtheriticum* (SDC). Cultures were incubated under aerobic conditions with 5% CO^2^ at 37°C for 18 h. To account for the intrinsic heterogeneity among strains, each strain was tested in triplicate (Fig. 1)*.*

#### Quantitative assessment of *S. pneumoniae* growth

DNA was extracted from 400 µL of each of the four pneumococcal cultures using the eMAG platform (bioMerieux; Marcy-l’Étoile, France) with a final elution volume of 50 µL.

*S. pneumoniae* was quantified using a real-time PCR assay targeting the *lytA* gene following serial dilutions of 1:10 of the genomic extract. The selection of the *lytA* gene, along with its primers and probes, was employed according to the Centers for Disease Control and Prevention (CDC) guidelines. This gene is a specific target, making it an excellent marker for detecting the presence of this pathogen in clinical samples (34).

A calibration curve was established to correlate *S. pneumoniae* DNA concentration (ng/µL) with the cycle threshold (CT) values obtained from the real-time PCR. The calibration curves were generated from the extraction of genomic DNA from a Todd-Hewitt suspension of *S. pneumoniae* of each strain. The DNA concentration was measured using Qubit Fluorometric Quantification with serial 1:10 dilutions prepared from the range of 10 to 10^−6^ ng/µL (35). The resulting pneumococcal IVG values, initially quantified in ng/ µL, were converted to gc/µL using the median genome size of *S. pneumoniae*, which is 2.085 kbp (36) (Fig. 1).

### Statistical analyses

The effect of commensal bacteria on pneumococcal growth was considered according to microbial and clinical characteristics of pneumococcal infection: serotype invasiveness, healthy pneumococcal nasopharyngeal carriage or IPD status, and isolation site (blood or nasopharynx).

A linear mixed-effect regression model (LMM) was used to evaluate the impact of *D. pigrum* and *C. pseudodiphtheriticum* on *S. pneumoniae*’s growth. To normalize the distribution, the outcome variable, pneumococcal growth, underwent a log transformation. To account for the triplicates, the strain identifier and the strain replicate were used as random effects in the LMM. This statistical approach allows for control and measurement of the inherent variability in pneumococcal growth between strains (strain identifier) while also accounting for the variability within the triplicates (strain replicate nested within strains). The incorporation of these random effects ensures not biased results derived from fixed effects.

All non-collinear variables examined—the culture condition, serotype invasiveness, and site of isolation—were included in an initial LMM. To determine which variables should be used as a fixed effect, a stepwise approach from the initial LMM was used to iteratively remove variables, resulting in different models. The Akaike information criteria (AIC), restricted maximum likelihood (REML), and determination coefficient (R²) (37–39) were the three key adjustment criteria used in each step to assess model fit and identify the significant predictor variables. In addition, the likelihood ratio test was used to compare the models performed (40). This process led to the selection of the optimal LMM, LMM2.

To assess the collinearity among the fixed effect variables, the variance inflation factor (VIF) was used, and those with a VIF of >5 were deemed collinear (41). Associations between these variables were measured by estimated coefficient (β), and their significance was assessed by estimated marginal means. The 95% confidence intervals (CIs) were calculated, and statistical significance was determined by setting a *P*-value of 0.05.

All the statistical analyses were performed using the 4.3.1 version of R and RStudio software (42) using the *car* (43), *lme4* (44), *emmeans* (45), *lmerTest* (46), and *MumIn* (47) packages.

## RESULTS

### Pneumococcal strain’s features

During the study period (2016–2023), a total of 28 *S*. *pneumoniae* strains were selected for analysis; 18 were isolated from blood and 10 from nasopharyngeal samples (two from IPD patients and eight from healthy children). Twenty-four different serotypes and 27 different clones were detected in the 28 pneumococcal strains. A proportion of 60.71% of serotypes (*n* = 17) were classified as low invasive disease potential serotypes (Table 1)*.*

**TABLE 1:** Characteristics of pneumococcal strains included in this study^*a*^

Strain	Serotype	Invasiveness of pneumococcal serotypes	Clone	Year of isolation	Site of isolation	Health status
1	10A	LIPST	97	2015	NPA	Healthy
2	9N	LIPST	66	2016	NPA	IPD
3	6C	LIPST	4,310	2016	NPA	Healthy
4	6B	LIPST	13,321	2016	NPA	Healthy
5	15B	LIPST	1,262	2017	NPA	Healthy
6	20	LIPST	1,871	2017	NPA	Healthy
7	19F	LIPST	1,167	2017	NPA	Healthy
8	15A	LIPST	5,139	2018	NPA	Healthy
9	33F	HIPST	717	2018	NPA	Healthy
10	11A	LIPST	6,521	2018	NPA	IPD
11	5	HIPST	289	2017	Blood	IPD
12	2	LIPST	74	2019	Blood	IPD
13	6A	LIPST	NA	2019	Blood	IPD
14	23F	HIPST	277	2021	Blood	IPD
15	7F	HIPST	3,544	2021	Blood	IPD
16	1	HIPST	306	2023	Blood	IPD
17	22F	LIPST	433	2022	Blood	IPD
18	18C	HIPST	133	2023	Blood	IPD
19	14	HIPST	156	2023	Blood	IPD
20	19A	HIPST	695	2023	Blood	IPD
21	4	HIPST	205	2023	Blood	IPD
22	11A	LIPST	6,521	2023	Blood	IPD
23	8	HIPST	53	2023	Blood	IPD
24	12F	HIPST	3,377	2023	Blood	IPD
25	9N	LIPST	517	2023	Blood	IPD
26	3	HIPST	180	2023	Blood	IPD
27	17F	LIPST	392	2023	Blood	IPD
28	20	LIPST	15,084	2023	Blood	IPD

^*a*^ NA: Not available; IPD: invasive pneumococcal disease; NPA: nasopharyngeal aspirate; HIPST: high invasive disease potential serotypes; LIPST: low invasive disease potential serotypes.

### Descriptive results

All 28 pneumococcal strains were replicated three times, resulting in a total of 84 observations. The mean growth of pneumococcal isolates in monoculture (3.04 log_10_gc/µL; SD, 0.42) was higher compared with those co-cultured with *D. pigrum* (2.28 log_10_gc/µL; SD, 0.57), *C. pseudodiphtheriticum* (2.46 log_10_gc/µL; SD, 0.82), or both commensal species (2.07 log_10_cg/µL; SD, 0.78) (Table 2)*.* The mean growth of high invasive disease potential serotype (2.61 log_10_gc/µL; SD, 0.75) pneumococcal isolates was higher than low invasive disease potential serotypes (2.37 log_10_gc/µL; SD, 0.74). Similarly, pneumococcal isolates from blood samples showed a higher mean growth (2.53 log_10_gc/µL; SD, 0.72) compared with those from NPA (2.35 log_10_gc/µL; SD, 0.79) (Table 2).

**TABLE 2:** Mean bacterial culture growth of 28 pneumococcal strains according to different categorical variables under various culture conditions^*a*^

	Pneumococcal growth (log_10_ gc/µL) Mean + (sd)				
	*S. pneumoniae* (*S*)	*S. pneumoniae + D. pigrum* (*SD*)	*S. pneumoniae +* *C. pseudodiphtheriticum* (*SC*)	*S. pneumoniae + D. pigrum +* *C. pseudodiphtheriticum* (*SDC*)	Overall
Overall	3.041 (0.422)	2.279 (0.569)	2.459 (0.815)	2.071 (0.775)	
HIPST	3.106 (0.453)	2.447 (0.533)	2.614 (0.920)	2.287 (0.787)	2.613 (0.757)
LIPST	2.999 (0.400)	2.171 (0.570)	2.359 (0.732)	1.933 (0.742)	2.365 (0.738)
NPA	2.986 (0.475)	2.127 (0.624)	2.191 (0.884)	2.094 (0.762)	2.350 (0.787)
Blood	3.071 (0.391)	2.363 (0.523)	2.608 (0.742)	2.059 (0.789)	2.525 (0.729)

^*a*^ sd: standard deviation.

### Selection of optimal model

Three LMMs were conducted to identify which predictor variables of *S. pneumoniae* strains had an effect on their growth. The selection of the optimal model was based on a combination of statistical fit and the relevance of predictors. LMM2 presented nearly the lower AIC (553.737) and the lower REML (537.7) values, indicating better fit compared with LMM1 and LMM3. Moreover, the LMM1 had a predictor variable that was far from being statistically significant, supporting its exclusion. Although LMM2 did not present the greatest Marginal R^2^ value, the difference from the others is minimal. All the models had similar conditional values of conditional R². Although the differences between these values are small, there is a slight decrease in explanatory power from LMM1 to LMM3, suggesting that the impact of removing predictor variables was minimal but not entirely negligible. For these reasons, LMM2 provides a good balance between model fit and the inclusion of relevant predictors (Table 3 and Table S1).

**TABLE 3:** The effect of commensal bacteria on pneumococcal growth of 28 strains of *S. pneumoniae* using linear mixed-effect model^a^

Predictors	β	95%	SE	*P* value
Intercept	3.195	2.91, 3.479	0.146	*P* < 0.0001 ****
Culture condition				
SD vs S	−0.763	−0.941, –0.586	0.069	*P* < 0.0001 ****
SC vs S	−0.583	−0.761, –0.406	0.069	*P* < 0.0001 ****
SDC vs S	−0.971	−1.148, –0.793	0.069	*P* < 0.0001 ****
SD vs SC	−0.180	−0.357, –0.003	0.069	0.045 *
SDC vs SD	−0.207	−0.385, –0.030	0.069	0.0146 *
SDC vs SC	−0.387	−0.565, –0.210	0.069	*P* < 0.0001 ****
Invasiveness of serotype (LIPST vs HIPST)	−0.250	−0.601, 0.101	0.180	0.175
**Random Effects**				
σ^2^	0.197			
τ_00_ Replicate:Strain_ID	0.063			
τ_00_ Strain_ID	0.178			
ICC	0.550			
N _REPLICATE_	3			
N _STRAIN_	28			
Observations	336			

^a^ β: estimated coefficient; SE: standard error; σ2: residual variance; τ00: intercept variance; ICC: intraclass correlation coefficient; *P < 0.05; **P < 0.01; ***P < 0.001; ****P < 0.0001.

### Pneumococcal strain variability

The results of the LMM2 revealed considerable variability in pneumococcal growth among strains. The intraclass correlation coefficient (ICC) was 0.55, indicating that 55% of the total variability in growth could be attributable to differences between individual strains. The variance of the random effects between strains was 0.178, while the variance between replicates within the same strain was lower, 0.063 (Table 3)*.* These findings showed that a considerable portion of the observed variability in pneumococcal growth was accounted for by differences between individual strains rather than variation among replicates of the same strain.

### Inhibitory effect of *Dolosigranulum pigrum* and *Corynebacterium pseudodiphtheriticum* on pneumococcal growth

The results of LMM2 analysis provided strong evidence of the association between the culture condition and the pneumococcal growth, although pneumococcal strain variability was observed.

The presence of *D. pigrum* was significantly associated with a reduction in pneumococcal growth, as reflected by the estimate coefficient (β = −0.763, CI: −0.94 to −0.59, *P* < 0.0001). Similarity *C. pseudodiphtheriticum* exhibited a notable inhibitory effect (β = −0.583, CI: −0.76 to −0.41, *P* < 0.0001). Notably, the combined presence of both bacteria was strongly associated (β = −0.971, CI: −1.15 to −0.79, *P* < 0.0001) with pneumococcal growth decrease.

**Fig 2:**
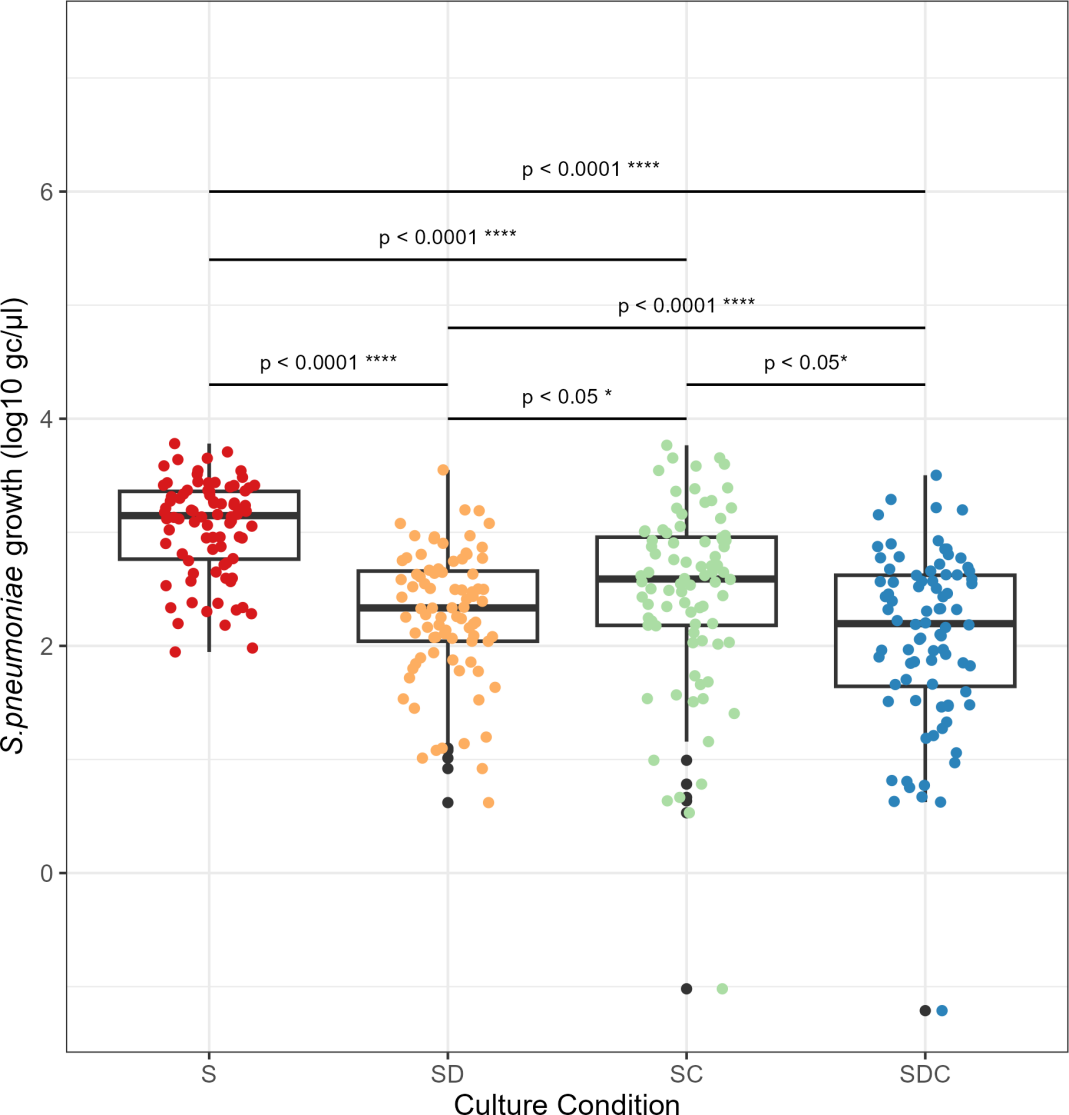
Pneumococcal growth under different culture conditions: cultured alone (red, labeled as S for *Streptococcus*), cultured with *D. pigrum* (orange, labeled as SD), cultured with *C. pseudodiphtheriticum* (light green, labeled as SC), and cultured with both commensal bacteria (dark blue, labeled as SDC). The data are represented by boxplots showing the median and interquartile range (IQR) of pneumococcal growth (measured in gc/μl after log transformation), along with individual data points (*n* = 338). Black points denote outliers. *P < 0.05; **P < 0.01; ***P < 0.001; ****P < 0.0001.

Even though *D. pigrum* and *C. pseudodiphtheriticum* showed an individual inhibitory effect on pneumococcal growth, their combined effect was even significantly stronger, with estimated coefficients of −0.207 (CI: −0.39 to −0.03, *P* < 0.01) and −0.387 (CI: −0.57 to −0.21, *P* < 0.0001) when compared with co-culture with *D. pigrum* alone or *C. pseudodiphtheriticum* alone, respectively (Fig. 2 and Table 3).

### Variation in pneumococcal growth according to strain features

**Fig 3:**
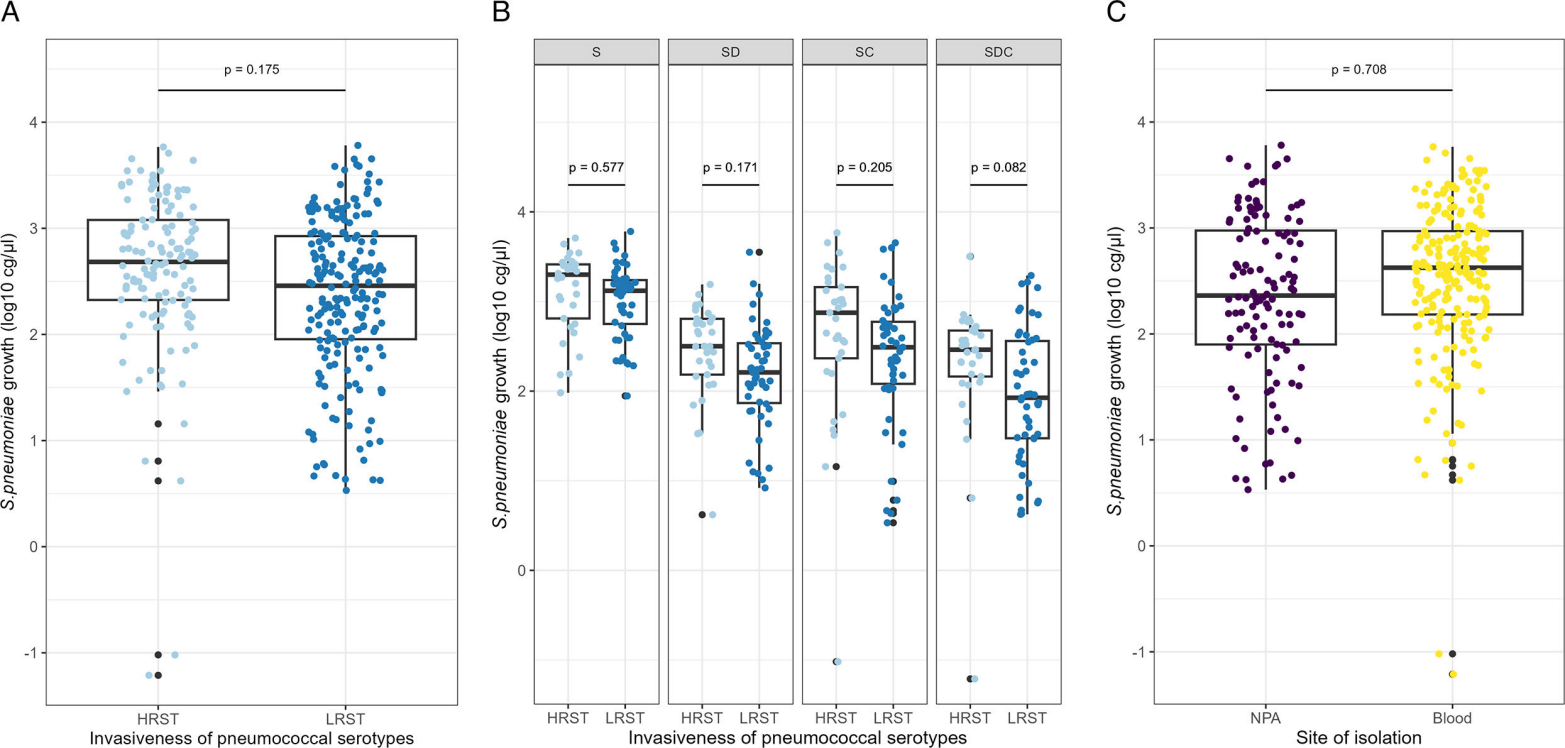
Pneumococcal growth according to strain features. (A) Invasiveness of pneumococcal serotypes, HIPST (light blue) vs. LIPST (dark blue). (**B**) Invasiveness of pneumococcal serotypes in different culture conditions. (**C**) Site of isolation, NPA (purple) vs blood (yellow). The data are represented by boxplots showing the median and interquartile range (IQR) of pneumococcal growth (measured in gc/μL after log-transformation), along with individual data points (*n* = 338). Black points denote outliers. *P < 0.05; **P < 0.01; ***P < 0.001; ****P < 0.0001.

In the LMM analysis, the site of isolation did not indicate any significant association with pneumococcal growth (β = −0.079, CI = −0.48 to 0.33, *P* = 0.708) (Fig. 3C and Table S1). Furthermore, pneumococcal growth appeared not to be generally affected by the invasiveness of pneumococcal serotype. Low invasive disease potential serotypes of pneumococcal isolates showed a slightly reduced growth, although not statistically significant, overall compared with high invasive disease potential serotypes (β = −0.250, CI = −0.60 to −0.10, *P* = 0.175) (Fig. 3A and Table 3). Moreover, a detailed subanalysis examining this predictor under different culture conditions revealed that low invasive disease potential serotype isolates showed a greater decrease in growth when they were cultured with both commensal bacteria (β = −0.355, CI = −0.76 to 0.05, *P* = 0.082) than when they were cultured alone, where the effect was minimal (β = −0.112, CI = −0.52 to 0.29, *P* = 0.577). However, this effect was not significant, indicating no clear association between these variables (Fig. 3B and Table S2).

## DISCUSSION

The present study evaluated the effect of two commensal bacteria from the nasopharyngeal microbiota on the growth of different pneumococcal strains as well as other characteristics of the strain itself, such as serotype invasiveness and the isolation site of the isolate, which may influence this effect.

The main findings of this study indicated that despite the variability among pneumococcal strains, the presence of those two commensal bacteria, alone or in combination, had a substantial impact on pneumococcal growth. Specifically, the growth of *S. pneumoniae* was significantly decreased in the presence of *D. pigrum,* supporting its potential interest as a candidate for inclusion in a probiotic formula. An ideal probiotic for the respiratory microbiota should be a non-pathogenic bacterium that is present as a natural carrier, capable of adhering to the epithelium and colonising the niche of key pathobionts. It also should have no cytotoxic effect on respiratory epithelial cells, resistance against horizontal gene transfer and mobile genetic elements, a low tendency for tissue invasion, and be susceptible to commonly used antibiotics (19, 26). Recent reviews of the literature highlight these characteristics of *D. pigrum*, supporting its potential as a probiotic candidate (19). Its presence in the upper respiratory tract suggests a natural ability to colonize the respiratory tract (25). In addition, recent studies have been conducted on animal models to evaluate the feasibility of using these species as probiotics (15, 16). Nevertheless, rare cases of infections have been reported with *D. pigrum,* which underscores the importance of thorough safety evaluations before its application as probiotics (48). Therefore, it is important to consider the regulatory challenges involved in developing probiotics in clinical applications.

Nonetheless, even with these promising features, the current published studies have been confined to very few strains and have not considered the extensive genetic diversity of *S. pneumoniae,* which consists of 100 serotypes and thousands of clones with different invasive disease potential (1, 2, 4, 49).

One of the key strengths of this study is the use of various strains of *S. pneumoniae* with different intrinsic characteristics, such as distinct serotypes, and isolated from different anatomical sites. This diversity in pneumococcal strains allowed for considering strain-specific differences in the pneumococcal growth in the analysis, providing stronger insights into potential interactions.

The analysis conducted on *S. pneumoniae* strain-specific variables revealed that the site of isolation (nasopharynx or blood) and serotype invasiveness did not significantly affect the antagonistic effect of commensal bacteria in pneumococcal growth. These findings suggest that the inhibition of *S. pneumoniae* is consistent regardless of its site of isolation and invasiveness, enhancing the capacity of *D. pigrum* as a potential probiotic. However, the limited number of strains emphasizes the importance of additional studies to validate this consistent antagonistic effect and thus better comprehend the underlying interactions.

The exact mechanisms by which *D. pigrum* exerts its observed antagonistic effect on pneumococcal growth remain unclear. One possible explanation is competition for nutrients, which are frequently a limiting factor for bacteria colonisation, such as *S. pneumoniae.* Therefore, *S. pneumoniae* experiences reduced nutritional availability when it coexists with other bacteria, which consequently restricts its growth (50).

Another factor could be the secretion of antimicrobial compounds, such as bacteriocins, which can function as a bactericidal or bacteriostatic agent against pathogens (23, 24). Specifically, it is suggested that *D. pigrum* may produce lantipeptides, a type of bacteriocin with notable antimicrobial activity that can disrupt bacteria’s cell walls, restricting pathogen proliferation (51–53). Thus, pneumococcal inhibition may be explained in part by *D. pigrum’s* antimicrobial characteristics. In order to comprehend the precise association between *D. pigrum* and *S. pneumoniae* and the role of bacteriocins in this instance, additional analyses are crucial.

*D. pigrum* as a LAB has the ability to generate lactate in the nasal microbiome. Lactate is an acid that can lower the environmental pH, creating unfavorable conditions for the optimal growth of *S. pneumoniae*, which grows in a more neutral pH (54). However, prior studies, such as those by Brugger et al., indicate that *D. pigrum*’s lactate production for itself is insufficient to fully inhibit pneumococcal growth (14). This suggests that additional inhibition mechanisms are taking place. In this context, it is crucial to further explore the combined effects of bacteriocin production and acidification as potential synergistic mechanisms in the inhibition of pneumococcal growth.

An interesting aspect of the results is the synergistic effect observed when another commensal bacterium was present. Specifically, the inhibition of *S. pneumoniae* growth was significantly greater when *D. pigrum* cooperated with *C. pseudodiphtheriticum*. Although both bacteria individually already showed inhibitory effects, their combined effect is significantly stronger. This suggests that the interaction between *D. pigrum* and *C. pseudodiphtheriticum* enhances the reduction of pneumococcal growth. This observation could be indicative of a synergistic interaction between these two commensal bacteria, which aligns with the study of Brugger et al. In this regard, *D. pigrum* is auxotrophic for certain nutrients, especially for amino acids, and it is hypothesized that *C. pseudodiphtheriticum* may provide these nutrients, enhancing their mutual inhibitory effects on *S. pneumoniae* (14, 55, 56). A synergistic effect of bacteriocins from different bacterial isolates cannot be ruled out either.

Some limitations should be noted in the study. First, the study focused on the effect of two specific commensal species on pneumococcal growth. However, the nasopharyngeal microbiota includes a complex network of bacterial interactions that could also influence pneumococcal growth. This study opens a new line of research into the interactions between pathobionts and specific commensal bacteria within the microbiota. Second, only a single strain of *D. pigrum* and *C. pseudodiphtheriticum* was used for the *in vitro* study, which limits the generalizability of the results due to potential strain-specific variations. It is possible that other strains of these commensal bacteria may not have the same inhibitory effect on *S. pneumoniae*. Third, Todd-Hewitt medium, although widely used, does not fully replicate the complexities of the nasopharyngeal environment. Further studies could use more representative models of the *in vivo* environment. Also, the results obtained should be considered in the context of previous findings on the dynamics of bacterial inhibition, where the order of exposure between commensal and pathogenic bacteria plays a crucial role (57). In this study, a bacterial invasion scenario, where a pathogen attempts to colonize a pre-existing respiratory microbiota, was simulated by adding *S. pneumoniae* to a previously grown culture of both commensal bacteria. Future studies could explore more scenarios to determine to what extent the order affects the ability of commensal bacteria to inhibit respiratory pathogens.

Furthermore, a few of the observed associations did not reach statistical significance, which could be explained by the limited sample size. Future research should consider increasing the sample size to improve accuracy. Moreover, future studies should also incorporate genomic, metabolomics, transcriptomic, and phenotypic studies to elucidate the possible mechanism of inhibition and the underlying mechanism of the observed synergistic effect of the two commensal bacteria.

These findings could have important implications for understanding microbial interactions within polymicrobial environments, particularly in the context of bacterial interference and competition within the respiratory tract. The observed inhibitory effects suggest that these commensal bacteria may play a protective role in mitigating pneumococcal replication. The antagonistic effect of these commensal bacteria on *S. pneumoniae* replication supports the potential protective factor of a healthy nasopharyngeal microbiota against IPD and underscores the potential of these microorganisms as promising probiotic candidates.

## SUPPLEMENTAL MATERIAL

Supplemental material spectrum.00138-25-s0001.docx Tables S1 and S2.

ASM does not own the copyrights to Supplemental Material that may be linked to, or accessed through, an article. The authors have granted ASM a non-exclusive, world-wide license to publish the Supplemental Material files. Please contact the corresponding author directly for reuse.
