# Supplementary material for: Synergistic inhibition of pneumococcal growth by Dolosigranulum pigrum and Corynebacterium pseudodiphtheriticum: insights into nasopharyngeal microbial interactions
Source: Microbiol Spectr. 2025 May 30;13(7):e00138-25. doi: 10.1128/spectrum.00138-25 (PMC12211077; doi:10.1128/spectrum.00138-25)
Supplement: Supplemental material — Tables S1 and S2. [file spectrum.00138-25-s0001.docx]

**Table S1: Comparison of the various linear mixed-effect models performed**

| Predictors | LMM 1  (β+ 95% CI) | LMM 2  (β + 95% CI) | LMM 3  (β + 95% CI) |
| --- | --- | --- | --- |
| Intercept | 3.123 (2.662, 3.584) | 3.195 (2.910, 3.479) | 3.042 (2.848, 3.238) |
| Culture condition | | | |
| SD vs S | -0.763 (-0.897, -0.629) | -0.763 (-0.897, -0.629) | -0.763 (-0.897, -0.629) |
| SC vs S | -0.583 (-0.717, -0.449) | -0.583 (-0.717, -0.449) | -0.583 (-0.717, -0.449) |
| SDC vs S | -0.971 (-1.105, -0.836) | -0.971 (-1.105, -0.836) | -0.971 (-1.105, -0.836) |
| Invasiveness of serotype (LIPST vs HIPST) | -0.216 (-0.607, 0.176) | -0.250 (-0.601, 0.101) |  |
| Site of isolation (NPA vs Blood) | -0.079 (-0.478, 0.330) |  |  |
| **Adjustment criteria** | | | |
| AIC | 556.908 | 553.737 | 552.065 |
| REML | 538.9 | 537.7 | 538.1 |
| Marginal R^2^ | 0.248 | 0.25 | 0.23 |
| Conditional R^2^ | 0.667 | 0.662 | 0.658 |

LMM1: all the predictor variables included

LMM2: Site of isolation not included

LMM3: Site of isolation and Invasiveness of serotypes not included

**Table S2: Linear mixed-effect model to analyse the effect of Invasiveness of serotype in presence or absence of commensal bacteria to pneumococcal growth**

| Predictors | β | 95% CI | SE | p value |
| --- | --- | --- | --- | --- |
| Intercept | 3.111 | 2.806, 3.415 | 0.155 | p < 0.0001 **** |
| Culture condition | | | | |
| SD vs S | -0.662 | -0.877, -0.447 | 0.11 | p < 0.0001 **** |
| SC vs S | -0.495 | -0.710, -0.300 | 0.11 | p < 0.0001 **** |
| SDC vs S | -0.822 | -1.037, -0.607 | 0.11 | p < 0.0001 **** |
| Invasiveness of serotype (LIPST vs HIPST) | -0.112 | -0.642, 0.077 | 0.2 | 0.577 |
| Culture condition*Invasiveness of serotype | | | | |
| S: LIPST vs HIPST | -0.112 | -0.515, 0.291 | 0.2 | 0.577 |
| SD: LIPST vs HIPST | -0.278 | -0.681, 0.125 | 0.2 | 0.171 |
| SC: LIPST vs HIPST | -0.257 | -0.659, 0.146 | 0.2 | 0.205 |
| SDC: LIPST vs HIPST | -0.355 | -0.758, 0.047 | 0.2 | 0.082  **·** |
| **Random Effects** |  |  |  |  |
| σ^2^ | 0.197 |  |  |  |
| τ_00_ Replicate:Strain | 0.063 |  |  |  |
| τ_00_ Strain | 0.178 |  |  |  |
| ICC | 0.55 |  |  |  |
| N _REPLICATE_ | 3 |  |  |  |
| N _STRAIN_ | 28 |  |  |  |
| Observations | 338 |  |  |  |

β: Estimated Coefficient

SE: Standard Error

σ2 : Residual variance

τ00 : Intercept variance

ICC: Intraclass Correlation Coefficient
